# Catheter ablation or surgical therapy in moderate-severe tricuspid regurgitation caused by long-standing persistent atrial fibrillation. Propensity score analysis

**DOI:** 10.1186/s13019-020-01336-3

**Published:** 2020-09-29

**Authors:** Jiangang Wang, Songnan Li, Qing Ye, Xiaolong Ma, Yichen Zhao, Jie Han, Yan Li, Shuai Zheng, Kemin Liu, Meng He, Wen Yu, Junhui Sun, Xu Meng

**Affiliations:** 1 Department of Cardiac Surgery, Beijing Anzhen Hospital, Capital Medical University, No.2 Anzhen Road, Chaoyang District, Beijing, 100029 PR China; 2 Department of Cardiology, Beijing Anzhen Hospital, Capital Medical University, Beijing, China

**Keywords:** Tricuspid regurgitation, Atrial fibrillation, Outcomes

## Abstract

**Background:**

This study aimed to describe the mid-term outcomes of different treatments in patients with atrial fibrillation caused tricuspid regurgitation.

**Methods:**

A retrospective study of patients diagnosed as atrial fibrillation caused moderate-severe tricuspid regurgitation undergoing ablation (*n* = 411) were reviewed. The surgical cohort (*n* = 114) underwent surgical ablation and tricuspid valve repair; the catheter cohort (*n* = 279) was selected from those patients who had catheter ablation.

**Results:**

The estimated actuarial 5-year survival rates were 96.8% (95% CI: 92.95–97.78) and 92.0% (95% CI: 85.26–95.78) in the catheter and surgical cohort, respectively. Tethering height was showed as independent risk factors for recurrent atrial fibrillation and tricuspid regurgitation in both cohorts. A matched group analysis using propensity-matched was conducted after categorizing total patients by tethering height < 6 mm and ≥ 6 mm. Kaplan–Meier analysis showed in patients with tethering height < 6 mm, there were no differences in survival from mortality, stroke, recurrent atrial fibrillation and tricuspid regurgitation between two groups. In patients with tethering height ≥ 6 mm, there were significantly higher cumulative incidence of stroke (95% CI, 0.047–0.849; *P* = 0.029), recurrent atrial fibrillation (95% CI, 0.357–09738; *P* = 0.039) and tricuspid regurgitation (95% CI, 0.359–0.981; *P* = 0.042) in catheter group.

**Conclusions:**

Atrial fibrillation caused tricuspid regurgitation resulted in less leaflets coaptation, which risk the recurrence of atrial fibrillation and tricuspid regurgitation. Patients whose tethering height was less than 6 mm showed satisfying improvement in tricuspid regurgitation with the restoration of sinus rhythm after catheter ablation. However, in patients with severe leaflets tethering, the results favored surgical over catheter.

## Introduction

Functional tricuspid regurgitation (TR) with a normal structural tricuspid valve (TV) leaflets and chordae often occur secondary to long-standing persistent atrial fibrillation (LSPAF) [1, 2]. This causes abnormalities of surrounding or supporting structures, such as annular dilatation and right ventricular (RV) dysfunction, promoting dilation of atrioventricular valve annuli that ultimately results in moderate-severe TR AF (TRAF) [3]**.** Moreover, increased mortality among patients with TRAF, regardless of only class 1 indication for TV repair during concomitant left heart surgery (Level of Evidence: C), has raised interest in various therapeutic strategies [4].

Catheter ablation targeting pulmonary veins (PVs) isolation has become the common treatment for atrial fibrillation (AF). To prevent recurrences in patients with LSPAF, isolation of PVs alone may not be sufficient. A fixed approach is developed for catheter ablation of LSPAF, which consists of bilateral circumferential pulmonary vein antrum isolation (PVAI) (‘2C’) and three linear ablation sets (‘3 L’) [5]**.** Functional atrioventricular valvular regurgitation caused by AF could eliminate by catheter ablation, and the negative atrial remodeling induced by valvular regurgitation could also be reversed, decreasing the recurrence of AF after ablation [6, 7]**.** However, it is not known whether the AF-caused moderate-severe TR could eliminate after ‘2C3L’ procedure.

Moreover, the development of open surgical ablation of AF has led to their widespread use during cardiac operations [8–11]**.** Surgical ablation concomitant with TV repair, therefore, might be the optimal treatment for the patients with AF-caused moderate-severe TR. However, the progression of TR and rhythm status after surgical treatment has not been comprehensively evaluated.

Therefore, we have conducted a retrospective cohort study to evaluate the differences between the catheter and surgical treatment in patients with LSPAF caused moderate-severe TR in the mid-term follow-up. Moreover, by assessing the heart rhythm status, and TV and RV functions before and after different treatments, the contributions of these treatments to AF and TR were detected.

## Methods

### Patient selection

The institutional review board at the Beijing Anzhen Hospital, Capital Medical University has approved the study. All patients referred for catheter or surgical ablation for LSPAF with moderate-severe TR between January 2008 and December 2013 were considered eligible for inclusion. Patients underwent surgical ablation concomitant with TV repair were included in the surgical cohort. The catheter cohort included patients who underwent ‘2C3L’ ablation. The exclusion criteria were: 1) previous percutaneous coronary intervention or cardiac surgery; 2) patients with more than moderate mitral regurgitation; 3) patients with an ejection fraction of < 40%.

### Catheter ablation and surgical procedure

**Fig. 1:**
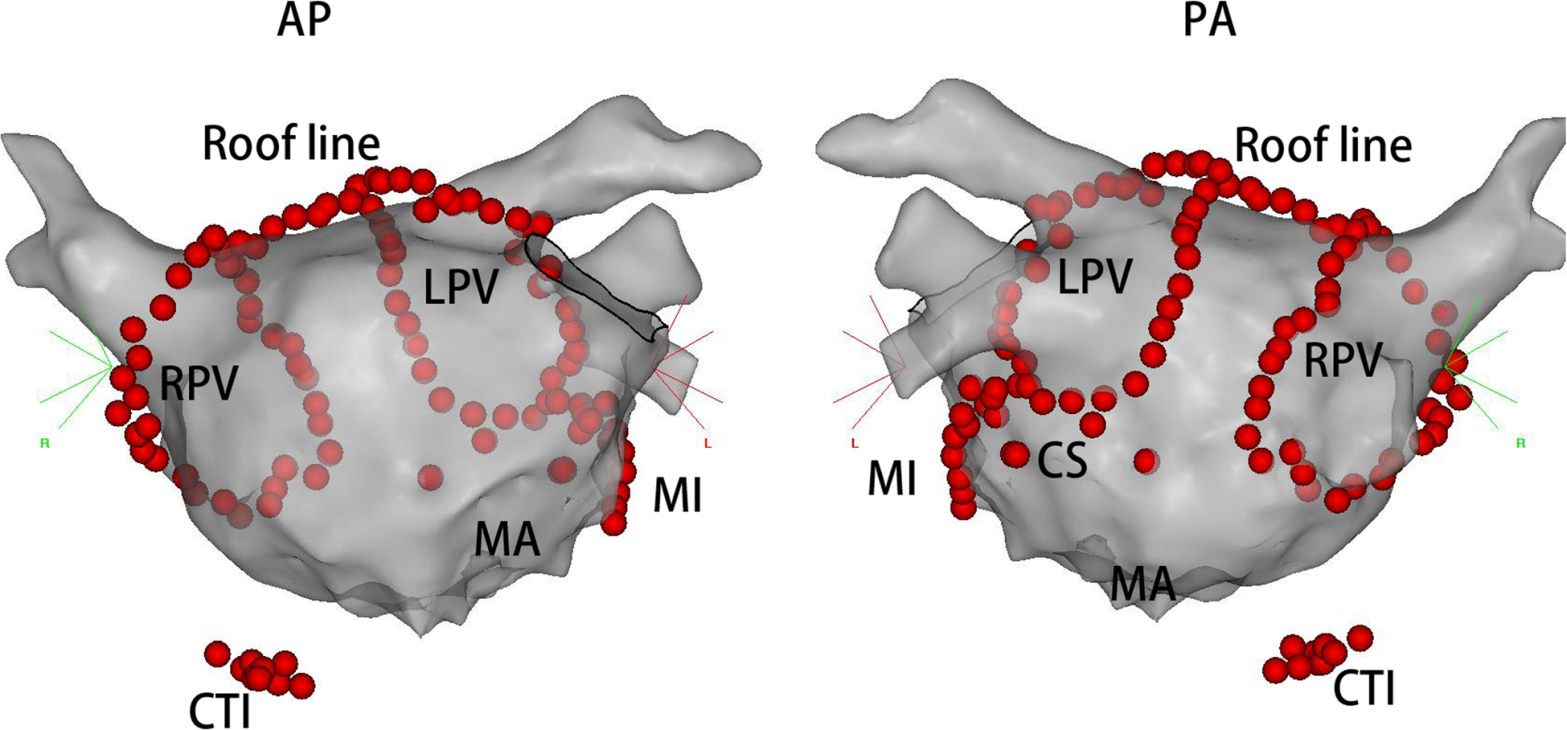
Example of a patient in catheter cohort undergoing ablation by using the ‘2C3L’ technique. Typical lesion set of a ‘2C3L’ procedure shown on a shell of the left atrium constructed by a 3D electro-anatomical mapping (CARTO). The red dots represented endocardial ablation. AP, anteroposterior; CS, coronary sinus; CTI, cavo-tricuspid isthmus; LPV, left pulmonary vein; MA, mitral annulus; MI, mitral isthmus; PA, posteroanterior; RPV, right pulmonary vein

A fixed approach for ablation of long-standing persistent AF was developed, which consists of bilateral circumferential PVAI (‘2C’) and three linear ablation sets (‘3 L’) (Fig. 1). Linear ablation is empirically applied across the mitral isthmus (MI), the left atrial (LA) roof, and the cavo-tricuspid isthmus (CTI). Cardioversion was performed if AF persisted or converted into an organized atrial tachyarrhythmia (OAT) following the initial pulmonary vein (PV) antrum and linear ablations. If cardioversion failed or atrial fibrillation (AF) has immediately relapsed, amiodarone was intravenously administered before repeat cardioversion. After the restoration of sinus rhythm (SR), radiofrequency applications were applied, and if needed, to close the gaps along with the circumferential lesions to achieve pulmonary vein antrum isolation **(**PVAI**)** was later verified by a circular mapping catheter. Similarly, conduction across the LA roofline, the MI line, and the CTI line were evaluated by using differential pacing manoeuvers. Also if necessary, the ridge between the LA appendage (LAA) and the left superior PV was targeted. Further ablations were conducted to achieve complete linear block as previously reported. If an OAT occurred after PVAI and documented the blockage of the lines, this tachycardia was then ablated with guidance of 3D electroanatomic mapping and conventional electrophysiological manoeuvres. If AF relapsed after PVAI and linear block across the LA roof, MI and CTI lines were achieved, then the superior vena cava (SVC) was isolated following cardioversion. Procedural endpoint included the isolation of PV antrum and complete conduction of block across the three ablation lines.

Patients who experienced arrhythmia recurrence after the blanking period were treated with cardioversion, medication, or repeated ablation at the patient’s and physician’s discretion. A redo procedure followed the ‘2C3L’ strategy where the conduction gaps were targeted, and clinical OATs were also mapped and ablated; however, complex fractionated atrial electrograms (CFAEs) ablation were not performed during the repeat procedure. After completing the PVAI and blockage across the three lines (LA roof, MI, and CTI), burst pacing at 200 ms was applied from proximal coronary sinus (CS) to induce tachycardia. Only the traceable OAT further underwent ablation, and the endpoint included was that the targeting OAT became non-inducible. Otherwise, the procedure was terminated by cardioversion.

#### Surgical ablation procedure

Ablation was carried out with bipolar Cardioablate (Medtronic, Minneapolis, MN) or an Atricure clamp (Atricure, West Chester, OH) as we described previously [12]. After cross-clamping the aorta, the heart was arrested, and the LA was accessed through left atriotomy. The LA ablation line includes isolation of PVs and a connecting line was performed between both islands of PVs on the roof and a line from the left PVs to the posterior mitral annulus. The LAA was excised or excluded. Right atrial (RA) ablation line includes SVC to inferior vena cava; the lateral free-wall lesion completes the anterior-medial tricuspid valve annulus; medial free-wall lesion completes the anterior-medial TV annulus and CTI ablation. The procedure was considered complete with the ablation of the right appendage.

### Tricuspid valve repair

TV annuloplasty was subsequently performed with a Carpentier-Edwards MC3 ring (Edwards Lifesciences). The tricuspid ring was sized according to the combined surface area of the posterior and anterior tricuspid leaflets that are extended using a right-angle hook and implanted with simple, interrupted mattress sutures by sparing the septal annulus and conduction tissue in the region of the apex of the triangle of Koch.

#### Post-procedure management

Warfarin was given to maintain the international normalized ratio between 2.0 and 2.5 for the first 3 months to all patients. Patients who have no AF recurrence and a CHADS2 score < 2 stopped taking warfarin 3 months following the procedure. Unless contraindicated, patients received amiodarone or sotalol within 24 h of the procedure, which were discontinued at 3 months (blanking period).

#### Echocardiography

Comprehensive 2-dimensional (2D) echocardiography was performed with a commercially available system (IE33; Philips Medical Systems, Andover, MA). Standard 2D and Doppler echocardiographic images were acquired in the left lateral decubitus position using a phased-array transducer in the parasternal and apical views by experienced cardiac sonographers. Three consecutive cardiac cycles were recorded and stored for subsequent analysis. Left ventricle end-diastolic (LVEDD) and end-systolic dimensions (LVESD) were measured from parasternal acquisitions. Left ventricle volumes and left ventricle ejection fraction (LVEF) were calculated using Simpson’s biplane method according to the guidelines of the American Society of Echocardiography [13]. LA and RA areas were measured by planimetry at end-systole from the apical 4-chamber views. Left atrial volumes were measured by Simpson’s biplane method. Color flow was applied in the apical 4-chamber view to assess the severity of TR, which was then graded semiquantitatively based on a scale of 0 to 4, where 0, none or trace; 1+, jet area/atrial area < 10% (mild TR); 2+, jet area/atrial area 10 to 20% (moderate TR); 3+, jet area/atrial area 20 to 33% (moderate-severe TR); and 4+, jet area/atrial area > 33% (severe TR) [14]. Tethering height was measured by tracing between the atrial surface of the leaflets and the tricuspid annular plane at the time of maximal systolic closure. From the apical 4-chamber view, the RV end-systolic and end-diastolic areas were measured by planimetry by positioning the transducer to maximize the RV area and to include the RV apex. RV fractional area change (RVFAC) was used to determine the RV systolic function and was calculated by the following formula: FAC = ([diastolic area systolic area]/diastolic area) × 100% [15]. RV long-axis length and RV short-axis width at the midventricular level were measured as described by Matsunaga and Duran and used to calculate the end-diastolic RV sphericity index (RVSI) as previously described (RVSI = RV long-axis length/RV short-axis width) [16]. Systolic pulmonary artery pressure (sPAP) was measured by echocardiography using the modified Bernoulli equation on the transtricuspid continuous-wave Doppler signal, while adding pressure on RA. The TV tethering height and TV tethering area were measured by tracing between the atrial surface of the leaflets and the tricuspid annular plane at the end-systole.

### Patient follow-up

All patients had visits scheduled at 1, 3, and 12 months postoperatively and every 1 year thereafter. At each visit, the patient’s history, physical examination, chest X-ray, a 24-h Holter, and echocardiogram were performed and obtained. The earliest echocardiogram on which moderate or greater TR was indicated for each patient was used to designate recurrent TR.

#### Statistics

All continuous variables are presented as mean standard deviation (SD), categorical values are presented as percentages, and odds ratios are presented with 95% confidence intervals (CIs). Comparisons between surgical and catheter cohorts were done by *t* test for continuous variables, chi-square test for categorical variables. A univariate and multivariate Cox hazard regression analysis was used to identify predictors of survival and freedom from events. The optimal cut-off value of the parameters in predicting the recurrence was identified using receiver operating characteristic (ROC) curve analysis. The area under the ROC curve (AUC) was calculated and compared by the DeLong method. Variables selected to be tested on multivariate analysis included those with *P* < 0.1 on univariate analysis. To reduce the effect of potential confounding factors in subgroup analysis, propensity-score match (PSM) analysis was used. The analysis was performed by matching patients in the 2 groups at a 1:1 ratio, without replacement, by the nearest neighbor technique, using a caliper width equal to 0.2 of the SD of the logit of the propensity score. A competing risk regression model was used to assess AF recurrence and stroke with death as a competing event. All significance tests were 2-tailed, and *p* value of < 0.05 was considered to be statistically significant. All analyses were performed with Stata/SE version 15 (Stata Corporation, Lakeway Drive College Station, TX, USA).

## Results

### Patients

There was a total of 1916 patients who underwent first catheter or surgical ablation between January 2008 and December 2013. There were only 411 patients that met the study criteria after review. Of these, 132 were treated by surgical ablation and TV repair, and 114 of whom had no history of left-sided valve surgery were included as the surgical cohort. Of the remaining 279 patients who were treated with ‘2C3L’ catheter ablation were selected as the catheter cohort. Baseline characteristics were shown in Table 1.

**Table 1:** Baseline characteristics before and after propensity-score matching

	Pre-matching			Post-matching		
	Catheter *n* = 279	Surgical *n* = 114	SMD	Catheter *n* = 111	Surgical *n* = 111	SMD
Age, yrs	69.1 ± 4.2	68.3 ± 4.8	0.095	69.0 ± 4.7	68.8 ± 4.5	0.15
Male	153 (54.8)	60 (52.6)	0.209	60 (54.1)	58 (52.3)	−0.177
BMI, kg/m^2^	22.8 ± 2.3	22.7 ± 2.7	0.097	22.7 ± 2.5	22.7 ± 2.5	0.074
NYHA Class	2.3 ± 0.4	2.5 ± 0.6	0.027	2.3 ± 0.5	2.3 ± 0.5	0.026
Smoking	71 (25.4)	30 (26.4)	−0.158	28 (25.2)	29 (26.1)	−0.127
Diabetes	29 (10.4)	13 (11.4)	−0.202	12 (10.8)	12 (10.8)	0.049
Hypertension	61 (21.9)	27 (23.7)	−0.148	25 (22.5)	26 (23.4)	−0.196
Stroke	34 (12.2)	17 (14.9)	0.016	15 (13.5)	16 (14.4)	0
CAD	31 (11.1)	15 (13.2)	0.003	14 (12.6)	14 (12.6)	0.081
COPD	12 (4.3)	6 (5.3)	0.016	5 (4.5)	5 (4.5)	−0.203
CHADS2 Score	0.68 ± 1.32	0.70 ± 1.82	0.058	0.69 ± 1.19	0.70 ± 1.23	0.076
Euro SCORE	0.75 ± 1.02	0.79 ± 1.10	−0.175	0.76 ± 1.09	0.78 ± 1.12	−0.274
AF duration, years	2.5 ± 1.1	3.3 ± 1.6	0.169	2.5 ± 1.2	2.6 ± 1.3	0
Medication						
ACE I	51 (18.2)	22 (19.3)	0.009	21 (18.9)	21 (18.9)	0.155
ARB	27 (9.7)	13 (11.4)	−0.011	11 (10.0)	12 (10.8)	0.176
CCB	20 (7.1)	11 (9.6)	−0.007	9 (8.1)	10 (9.0)	−0.137
Beta-blocker	52 (18.6)	14 (12.3)	0.136	15 (13.5)	14 (12.6)	−0.127
Digitalis	78 (28.0)	22 (19.3)	0.144	24 (21.6)	22 (19.8)	−0.156
Diuretics	83 (29.7)	24 (21.1)	0.113	26 (23.4)	24 (21.6)	−0.121
Amiodarone	100 (35.8)	30 (26.3)	−0.421	32 (28.8)	30 (27.0)	−0.17
LVESD, mm	36.1 ± 10.1	37.5 ± 11.6	0.013	36.9 ± 11.5	37.1 ± 10.8	0.105
LVEDD, mm	52.1 ± 5.8	53.7 ± 6.8	−0.163	52.8 ± 6.1	53.0 ± 6.3	0.161
sPAP, mmHg	39.1 ± 9.2	40.7 ± 6.2	−0.184	39.8 ± 8.9	40.0 ± 7.1	0.125
LAD, mm	52.1 ± 11.7	53.6 ± 10.8	−0.011	52.6 ± 10.3	52.9 ± 10.9	−0.055
LVEF, %	60.1 ± 8.2	58.6 ± 9.2	−0.127	59.6 ± 8.9	59.1 ± 9.0	0.071
RAA, mm^2^	19.7 ± 5.1	20.9 ± 3.5	−0.164	20.0 ± 4.9	20.1 ± 4.9	0.219
RVSI	2.0 ± 0.2	2.0 ± 0.5	−0.162	2.0 ± 0.3	2.0 ± 0.3	0.165
RVFAC, %	43.1 ± 4.8	42.2 ± 6.7	−0.243	42.7 ± 4.3	42.4 ± 5.1	−0.042
Tethering height, mm	7.1 ± 3.6	8.9 ± 3.9	−0.294	7.9 ± 3.4	8.2 ± 3.7	0.017
Tethering area, cm^2^	2.8 ± 0.8	3.7 ± 0.7	0.217	2.9 ± 0.9	3.2 ± 0.5	0
TR EROA, cm^2^	0.8 ± 0.4	0.9 ± 0.8	−0.127	0.8 ± 0.5	0.8 ± 0.7	−0.157
TR VC, cm	1.0 ± 0.4	1.0 ± 0.7	−0.133	1.0 ± 0.2	1.0 ± 0.3	0.075
TR grade	3.4 ± 0.7	3.7 ± 0.5	0.022	3.5 ± 0.5	3.6 ± 0.2	−0.095
TAD, cm	36.5 ± 4.1	39.1 ± 5.2	0.003	37.9 ± 4.9	38.8 ± 5.0	0.081

*ACEI* Angiotensin-converting enzyme inhibitor, *AF* Atrial fibrillation, *ARB* Angiotensin receptor blocker; *BMI* Body mass index, *CAD* Coronary artery disease, *CCB* Calcium channel blocker, *COPD* Chronic obstructive pulmonary disease, *EROA* Effective regurgitant orifice area, *LAD* Left atrial diameter, *LVEF* Left ventricle ejection fraction, *LVEDD* Left ventricle end-diastolic dimension, *LVESD* Left ventricle end-systolic dimension, *NYHA* New York Heart Association, *RAA* Right atrial area, *RVFAC* Right ventricle fractional area change, *RVSI* Right ventricle sphericity index, *SMD* Standard mean diference, *sPAP* Pulmonary artery systolic pressure, *TAD* Tricuspid annulus diameter, *TR* Tricuspid regurgitation, *VC* Vena contracta

### Procedural outcomes

All patients successfully underwent the procedure, and there was no in-hospital mortality. In both cohorts, 100% PVs that are targeted for ablation demonstrated entrance and exit block at the conclusion of the procedure. Intra-procedural AF termination was more (Table 2). In catheter cohort, the mean procedural time, fluoroscopy time and radiofrequency time were 215 ± 38 min, 48 ± 11 min and 116 ± 24 min, respectively. In surgical cohort, the mean duration of cardiopulmonary bypass and aortic cross-clamping was 97 ± 24 min and 46 ± 20 min, respectively.

**Table 2:** Details of the procedures

Procedure parameters	Catheter cohort *n* = 279	Surgical cohort *n* = 114	*P* value
AF termination rate, n (%)	145 (51.9)	90 (78.9)	< 0.0001
PVs entrance and exit block, n (%)	279 (100)	114 (100)	NS
Cardiac tamponade, n (%)	2 (0.7)	0 (0)	NS
Reexploration for bleeding, n (%)	0 (0)	1 (0.9)	NS
AKI requiring dialysis, n (%)	0 (0)	1 (0.9)	NS
CABG	0	5 (4.4)	0.060

*AF* Atrial fibrillation, *AKI* Acute kidney injury, *CABG* Coronary artery bypass graft, *PVs* Pulmonary veins

### Outcomes during follow-up

**Fig. 2:**
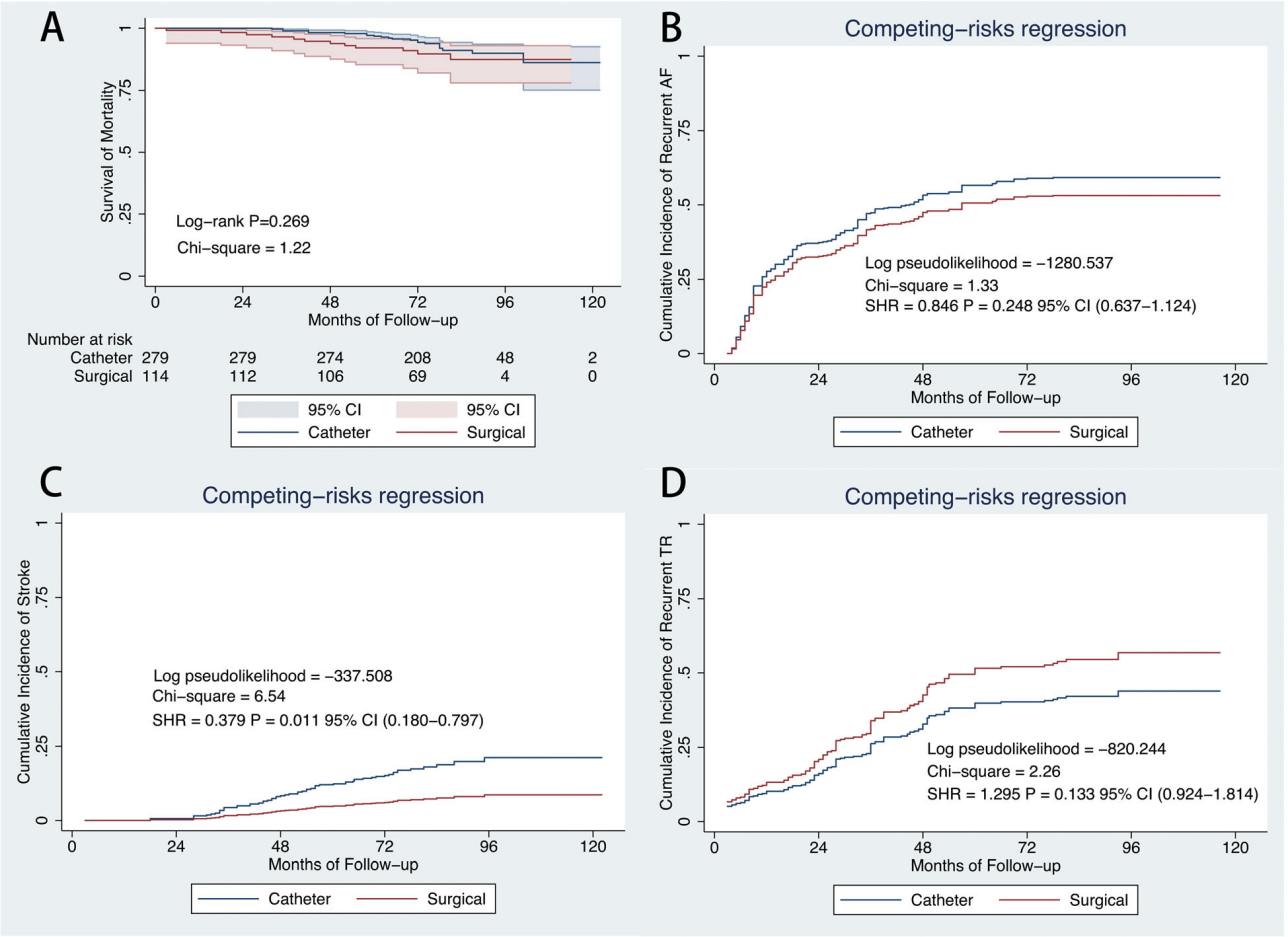
Outcomes in the two cohorts. Kaplan–Meier curves showing survival from mortality(**a**). Cumulative incidence curve of atrial fibrillation recurrence (**b**), stroke (**c**), and recurrent moderate-severe tricuspid regurgitation (**d**) by competing-risk regression. CI = confidence interval; SHR = sub-hazard ratio

All included patients have completed a 5-year clinical follow-up. After a mean follow-up period of 77 months, there were 22 and 12 deaths in the catheter and surgical cohort (Table 3). During the follow-up, only 1 patient required pacemaker implantation for sinus node dysfunction in surgical cohort. On Kaplan-Meier analysis, the estimated actuarial 5-year survival rates were 96.8% (CI: 92.95–97.78) and 92.0% (CI: 85.26–95.78) in the catheter and surgical cohort, respectively (Fig. 2a). In contrast to the surgical cohort, the catheter cohort had a higher risk of stroke (SHR: 0.3790; CI: 0.180–0.797; *P* = 0.011, Fig. 2c), but had a similar risk of recurrent AF (SHR: 0.8458; CI: 0.637–1.124; *P* = 0.248, Fig. 2b) and moderate-severe TR (SHR: 1.2950; CI: 0.924–1.814; *P* = 0.133, Fig. **2**d) after considering death as a competing risk.

**Table 3:** Causes of Death during Follow-up

	Catheter cohort *n* = 279	Surgical cohort *n* = 114	*P* value
Congestive heart failure, n (%)	10 (3.6)	9 (7.9)	0.373
Acute myocardial infarction, n (%)	1 (0.4)	0 (0)	NS
Stroke, n (%)	5 (1.8)	1 (0.9)	NS
Malignancy, n (%)	2 (0.7)	0 (00)	NS
Sudden cardiac death, n (%)	2 (0.7)	1 (0.9)	NS
Unknown cause, n (%)	2 (0.7)	1 (0.9)	NS

### Heart rhythm and echocardiography during follow-up

All patients in both cohorts had their complete Holter, and echocardiogram follow-up results. According to the rhythm status at follow-up, study population were divided into the patients with sinus rhythm (SR) and with recurrent AF in each cohort.

**Fig. 3:**
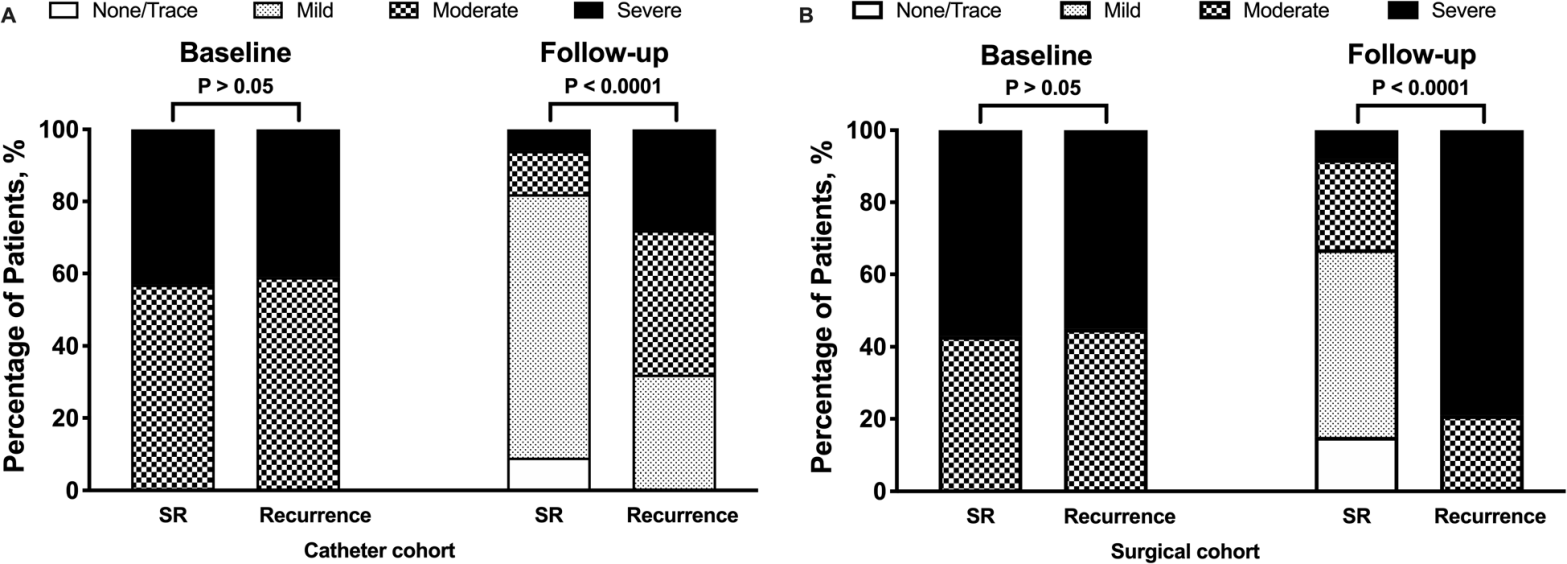
Change in tricuspid regurgitation grade change at baseline and follow-up in catheter cohort (**a**), and surgical cohort(**b**)

In catheter cohort, at baseline, both groups had similar percentages of patients with moderate-severe TR. At follow-up, 13% of patients with SR had trace or no TR, compared with 0% in the recurrent group, and 69% with SR had mild TR compared with 32% in the recurrent group. Only 18% of patients with SR still had moderate-severe TR at follow-up, compared with 68% in the recurrent group (*p* < 0.0001 for entire trend) (Fig. 3a). Moreover, SR group had a significant decrease in tethering height, tethering area, TR effective regurgitant orifice area (EROA), TR vena contracta (VC), TR grade and tricuspid annular dimension (TAD), with RV sphericity index (RVSI) and RV fractional area change (RVFAC) significantly increased at follow-up. And the recurrent group had a significant increase in right atrial (RA) area, TR EROA, TR grade and TAD, with RVSI and RVFAC significantly decreased at follow-up (Supplementary Table **1**).

In surgical cohort, at baseline, both groups had similar percentages of patients with moderate-severe TR. At follow-up, all patients with recurrent AF had moderate-severe TR at follow-up when compared with 33% in the SR group (*p* < 0.0001 for entire trend) (Fig. 3b). Moreover, SR group had a significant decrease in tethering height, tethering area, TR EROA, TR VC, TR grade and TAD, with RVSI and RVFAC significantly increased at follow-up. And the recurrent group had a significant increase in RA area and TAD, with RVSI and RVFAC significantly decreased at follow-up. (Supplementary Table 2).

#### Predictor for the recurrence of either AF or moderate-severe TR

**Fig. 4:**
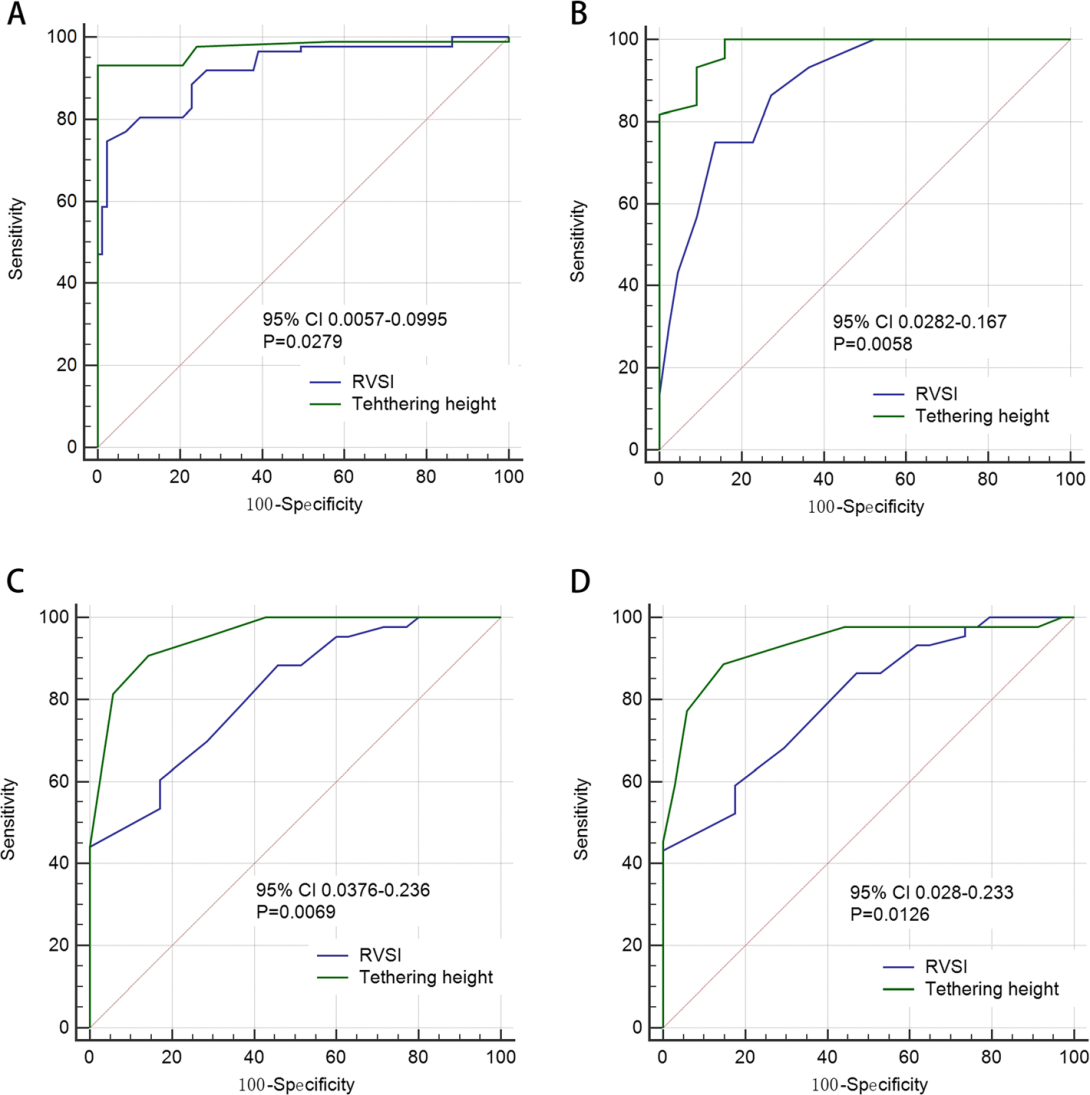
Comparing the areas under ROC curve of tethering height, and RVSI for the prediction of recurrent atrial fibrillation in catheter cohort (**a**) and surgical cohort (**c**); and for the prediction of recurrent moderate-severe tricuspid regurgitation in catheter cohort (**b**) and surgical cohort (**d**). CI = confidence interval

Multivariate Cox hazard regression analysis revealed that RVSI and tethering height as independent risk factors for the recurrence of either AF or moderate-severe TR in both cohorts (Tables 4 and 5). Next, the optimal cut-off value of the RVSI and tethering height in predicting the recurrence was identified using ROC curve analysis (Table 6). Interestingly, the tethering height showed a significantly better predictive performance than RVSI in the recurrence of either AF or moderate-severe TR in both cohorts (Fig. 4).

**Table 4:** Predictors of recurrence of AF in catheter and surgical cohorts: univariate and multivariate analyses

		Univariate		Multivariate	
		HR (95% CI)	*P* value	HR (95% CI)	*P* value
Age (per 10 years)	Catheter	3.12 (1.32–6.72)	0.048	2.90 (0.75–5.65)	0.184
	Surgical	3.34 (1.49–6.49)	0.020	3.01 (0.86–5.76)	0.152
CHADS2 Score	Catheter	4.12 (0.76–9.06)	0.079	3.17 (0.98–8.42)	0.801
	Surgical	3.39 (0.59–6.49)	0.077	2.50 (0.73–4.21)	0.199
AF duration, months	Catheter	4.02 (1.16–8.89)	0.014	3.41 (0.94–6.19)	0.461
	Surgical	4.86 (1.09–8.48)	0.037	3.71 (0.29–6.48)	0.303
LAD, mm	Catheter	1.05 (0.18–2.59)	0.097	0.97 (0.25–1.62)	0.344
	Surgical	1.46 (0.59–2.51)	0.065	1.10 (0.37–1.75)	0.290
RAA, mm^2^	Catheter	2.40 (1.50–5.26)	0.021	1.01 (0.32–3.39)	0.053
	Surgical	3.50 (1.69–8.03)	0.015	2.98 (0.79–8.23)	0.815
RVSI	Catheter	3.20 (1.20–7.13)	0.007	3.11 (1.69–6.76)	0.023
	Surgical	2.94 (1.63–6.60)	0.002	3.01 (1.27–5.23)	0.004
RVFAC, %	Catheter	3.01 (1.40–5.38)	0.021	1.31 (0.35–2.81)	0.076
	Surgical	1.47 (1.04–3.19)	0.016	1.19 (0.23–2.69)	0.110
Tethering height, mm	Catheter	5.32 (1.79–10.04)	0.017	4.06 (2.15–5.69)	0.011
	Surgical	4.62 (1.58–9.83)	0.011	3.82 (1.92–5.42)	0.035
Tethering area, cm^2^	Catheter	1.52 (1.06–4.25)	0.008	0.97 (0.30–1.46)	0.144
	Surgical	2.50 (1.66–6.39)	0.033	1.53 (0.73–2.21)	0.178
TR EROA, cm^2^	Catheter	3.53 (1.39–7.30)	0.028	1.95 (0.78–5.88)	0.082
	Surgical	2.49 (1.37–6.38)	0.025	1.85 (0.05–3.23)	0.121
TR VC, cm	Catheter	2.39 (0.35–6.59)	0.078	2.52 (0.50–5.04)	0.127
	Surgical	2.28 (0.18–6.18)	0.082	1.43 (0.26–5.37)	0.262
TR grade	Catheter	2.05 (1.58–5.33)	0.019	1.97 (0.69–3.26)	0.099
	Surgical	1.95 (1.01–5.21)	0.037	2.29 (0.98–3.58)	0.194
TAD, cm	Catheter	2.54 (1.19–5.95)	0.016	1.90 (0.76–4.58)	0.415
	Surgical	2.11 (1.57–5.52)	0.039	1.66 (0.52–4.34)	0.555

*AF* Atrial fibrillation, *CI* = CI Confidence interval, *EROA* Effective regurgitant orifice area, *LAD* Left atrial diameter, *RAA* Right atrial area, *RVFAC* Right ventricle fractional area change, *RVSI* Right ventricle sphericity index, *TR* Tricuspid regurgitation, *VC* Vena contracta

**Table 5:** Predictors of recurrence of TR in catheter and surgical cohort: univariate and multivariate analyses

		Univariate		Multivariate	
		HR (95% CI)	*P* value	HR (95% CI)	*P* value
Age (per 10 years)	Catheter	2.98 (1.44–5.82)	0.051	4.30 (0.50–8.30)	0.092
	Surgical	2.11 (1.69–6.36)	0.065	3.83 (0.94–7.69)	0.210
AF duration, months	Catheter	2.90 (1.04–4.86)	0.016	2.01 (0.73–4.29)	0.450
	Surgical	2.19 (1.71–6.94)	0.044	2.38 (0.83–5.12)	0.167
LAD, mm	Catheter	3.72 (0.50–8.61)	0.085	3.51 (0.94–7.23)	0.369
	Surgical	2.57 (0.98–5.18)	0.078	4.27 (0.63–6.17)	0.137
RAA, mm^2^	Catheter	4.63 (1.74–9.56)	0.042	4.36 (0.52–8.05)	0.254
	Surgical	4.22 (0.91–8.52)	0.051	5.54 (0.10–7.24)	0.080
RVSI	Catheter	3.63 (1.62–8.30)	0.004	3.13 (1.91–6.41)	0.009
	Surgical	3.18 (1.50–7.88)	0.005	3.21 (1.99–6.49)	0.015
RVFAC, %	Catheter	3.16 (1.43–7.76)	0.039	3.03 (0.84–4.47)	0.065
	Surgical	3.05 (0.93–6.36)	0.060	2.31 (0.12–3.75)	0.063
Tethering height, mm	Catheter	3.16 (1.17–7.30)	0.008	3.00 (1.69–5.56)	0.004
	Surgical	3.14 (1.35–8.38)	0.001	4.01 (2.71–6.58)	0.009
Tethering area, cm^2^	Catheter	2.60 (0.92–4.68)	0.079	1.38 (0.52–2.93)	0.063
	Surgical	1.49 (1.74–3.65)	0.028	1.46 (0.89–3.30)	0.084
TR EROA, cm^2^	Catheter	2.96 (0.61–6.56)	0.058	1.33 (0.84–4.62)	0.082
	Surgical	1.56 (0.92–5.53)	0.059	1.11 (0.61–4.39)	0. 065
TR VC, cm	Catheter	2.55 (0.86–5.60)	0.073	3.53 (0.84–7.55)	0.096
	Surgical	3.32 (0.72–7.96)	0.092	3.21 (0.50–7.19)	0.365
TR grade	Catheter	3.08 (1.47–7.80)	0.032	3.17 (1.17–7.37)	0.102
	Surgical	3.1 (0.92–6.52)	0.072	2.68 (1.48–6.04)	0.068
TAD, cm	Catheter	3.61 (0.74–7.17)	0.098	2.79 (0.50–6.88)	0.208
	Surgical	3.96 (0.68–9.47)	0.071	3.01 (0.32–8.01)	0.628

*AF* Atrial fibrillation, CI = CI Confidence interval, *EROA* Effective regurgitant orifice area, *LAD* Left atrial diameter, *RAA* Right atrial area, *RVFAC* Right ventricle fractional area change, *RVSI* Right ventricle sphericity index, *TR* Tricuspid regurgitation, *VC* Vena contracta

**Table 6:** ROC curve analysis of RVSI and tethering height in predicting the recurrence of AF and moderate-severe TR

		AF Recurrence							TR Recurrence						
		AUC	95% CI	Cutoff value	Sensitivity%	95% CI	Specificity%	95% CI	AUC	95% CI	Cutoff value	Sensitivity%	95% CI	Specificity%	95% CI
Catheter cohort	RVSI, mm	0.817	0.713–0.895	1.85	53.5	0.377–0.682	82.9	0.664–0.934	0.777	0.647–0.876	1.85	72.7	0.582–0.837	93.2	0.818–0.977
	Tethering height, mm	0.946	0.878–0.987	6	86.4	0.727–0.948	72.7	0.582–0.850	0.960	0.871–0.994	6	75.0	0.597–0.868	86.4	0.727–0.948
Surgical cohort	RVSI, mm	0.853	0.775–0.931	1.90	91	0.769–0.982	80	0.631–0.916	0.883	0.815–0.952	1.90	89.6	0.808–0.946	92.5	0.837–0.968
	Tethering height, mm	0.925	0.867–0.984	8	95.5	0.845–0.999	84.1	0.699–0.934	0.981	0.960–1.000	8	93.2	0.813–0.986	91.0	0.783–0,975

*AF* Atrial fibrillation, *AUC* Area under the receiver operating characteristic curve, *CI* Confidence interval, *ROC* Receiver operating characteristic, *RVSI* Right ventricle sphericity index, *TR* Tricuspid regurgitation

**Fig. 5:**
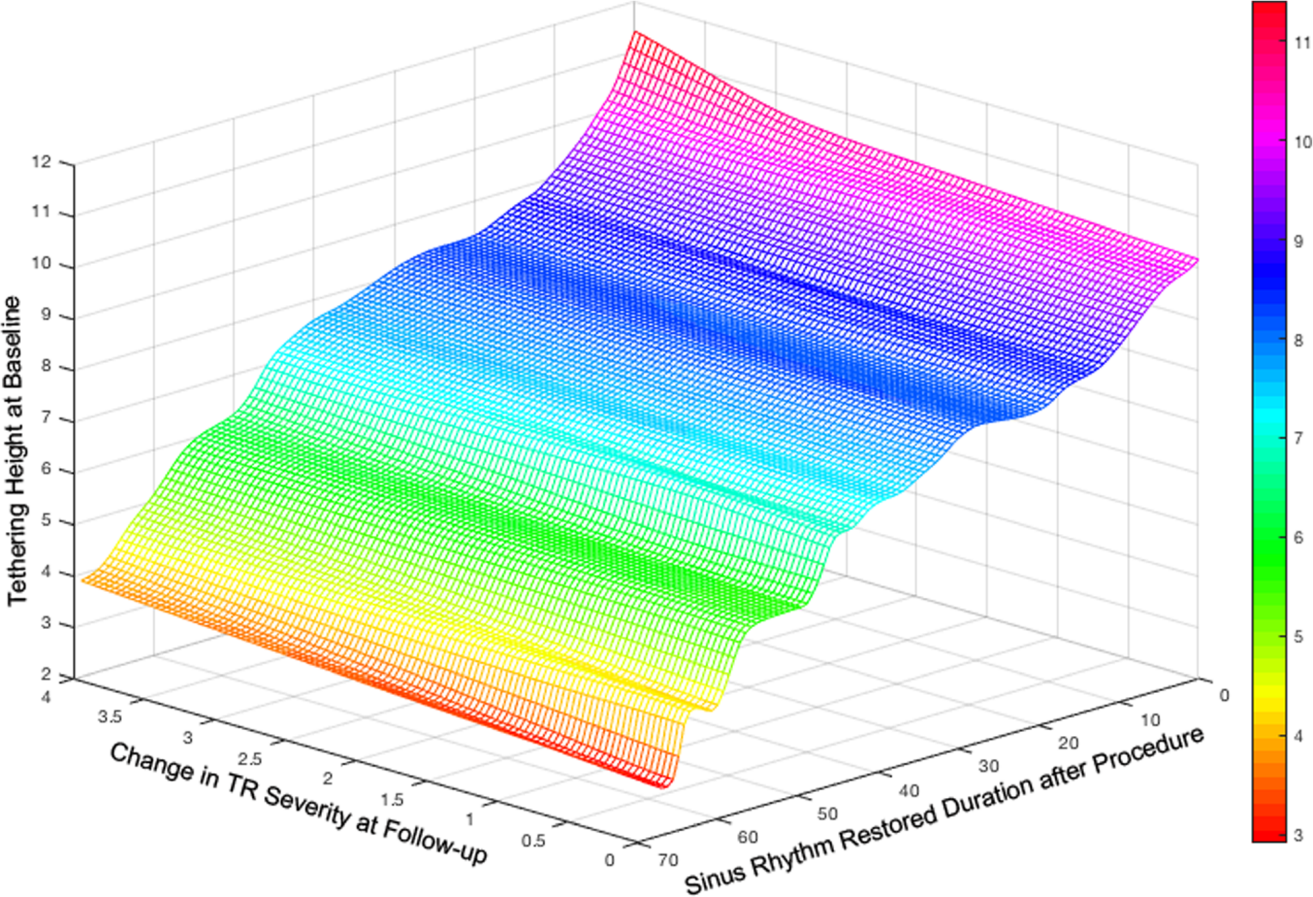
Relation between basal tethering height, change in tricuspid regurgitation severity at follow-up and sinus rhythm restored duration after the procedure

According to the merits of tethering height mentioned above, we made a 3-D plot graph to demonstrate the relation between basal tethering height, change in TR severity at follow-up and SR restored duration after the procedure in total cohort (Fig. 5). It showed the higher the basal tethering height was, the more changes in TR severity at follow-up were, and the less SR restored duration after the procedure was.

#### Propensity-score match analysis between two cohorts

**Fig. 6:**
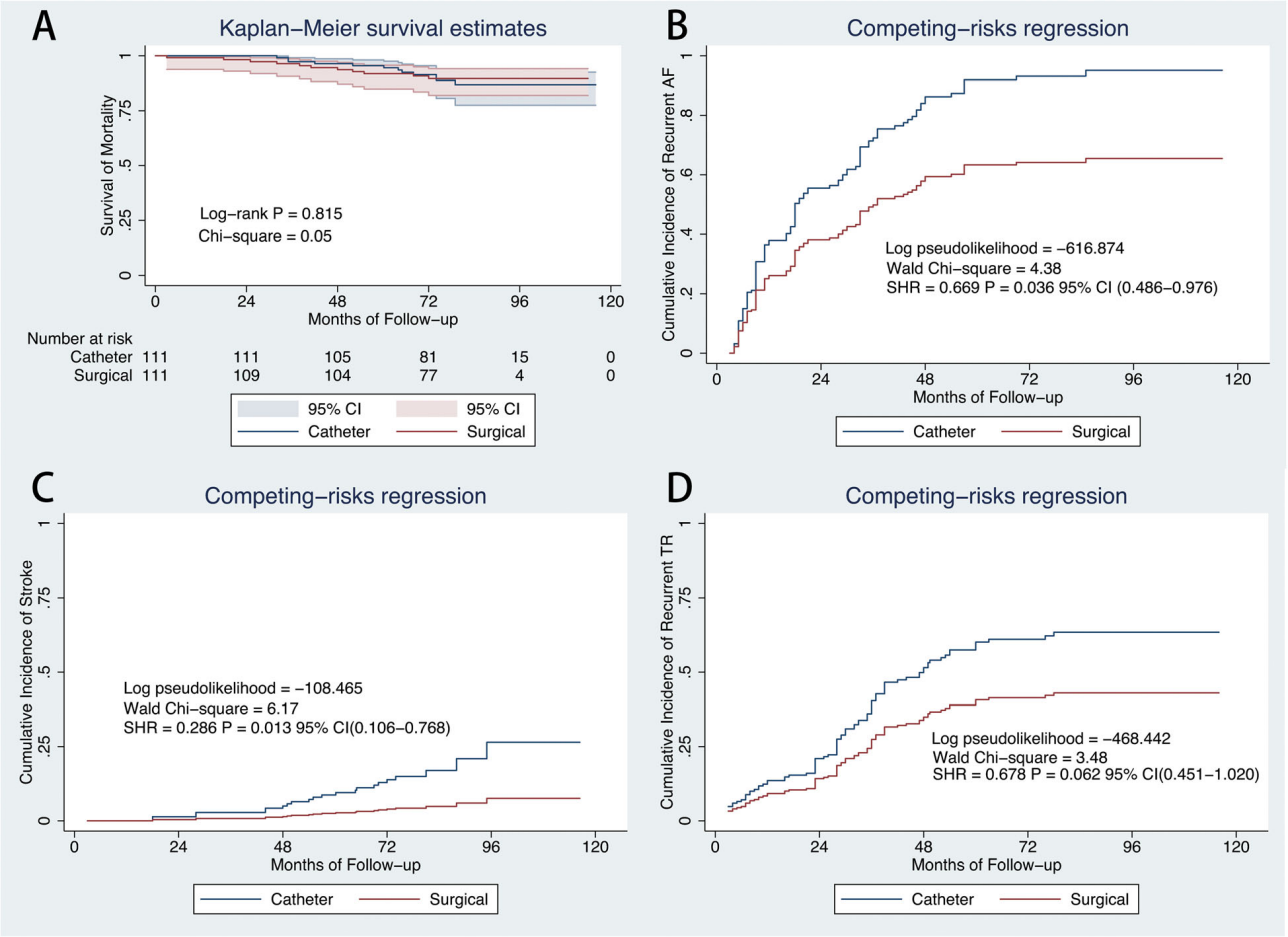
Comparison of outcomes in the PSM cohort. Kaplan–Meier curves showing survival from mortality(**a**). Cumulative incidence curve of atrial fibrillation recurrence (**b**), stroke (**c**), and recurrent moderate-severe tricuspid regurgitation (**d** by competing-risk regression. CI = confidence interval; SHR = sub-hazard ratio

**Fig. 7:**
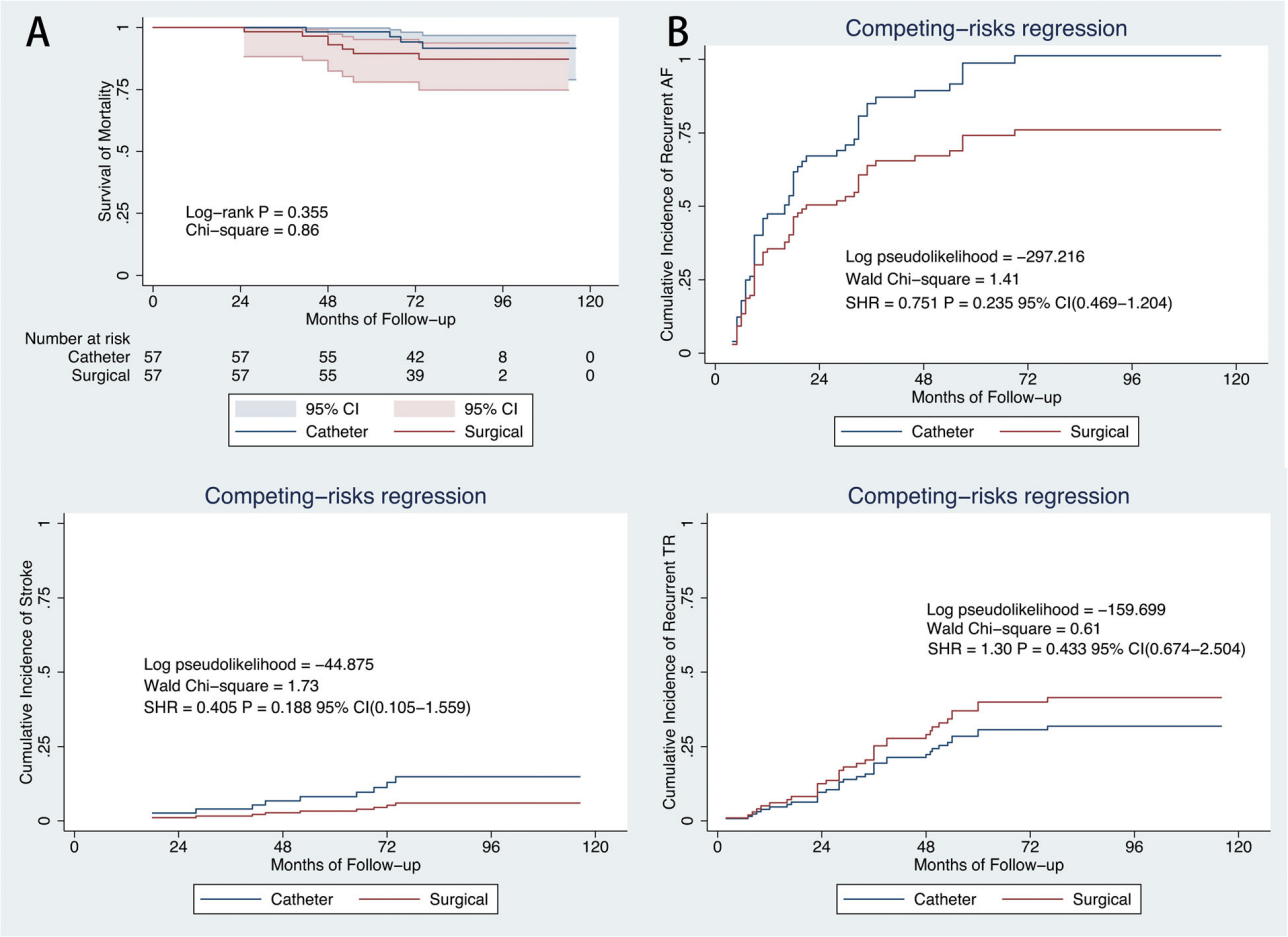
Comparison of outcomes in subgroups with tethering height < 6 mm after PSM. Kaplan–Meier curves showing survival from mortality(**a**). Cumulative incidence curve of recurrent atrial fibrillation (**b**), stroke (**c**), and recurrent moderate-severe tricuspid regurgitation (**d** by competing-risk regression. CI = confidence interval; SHR = sub-hazard ratio

**Fig. 8:**
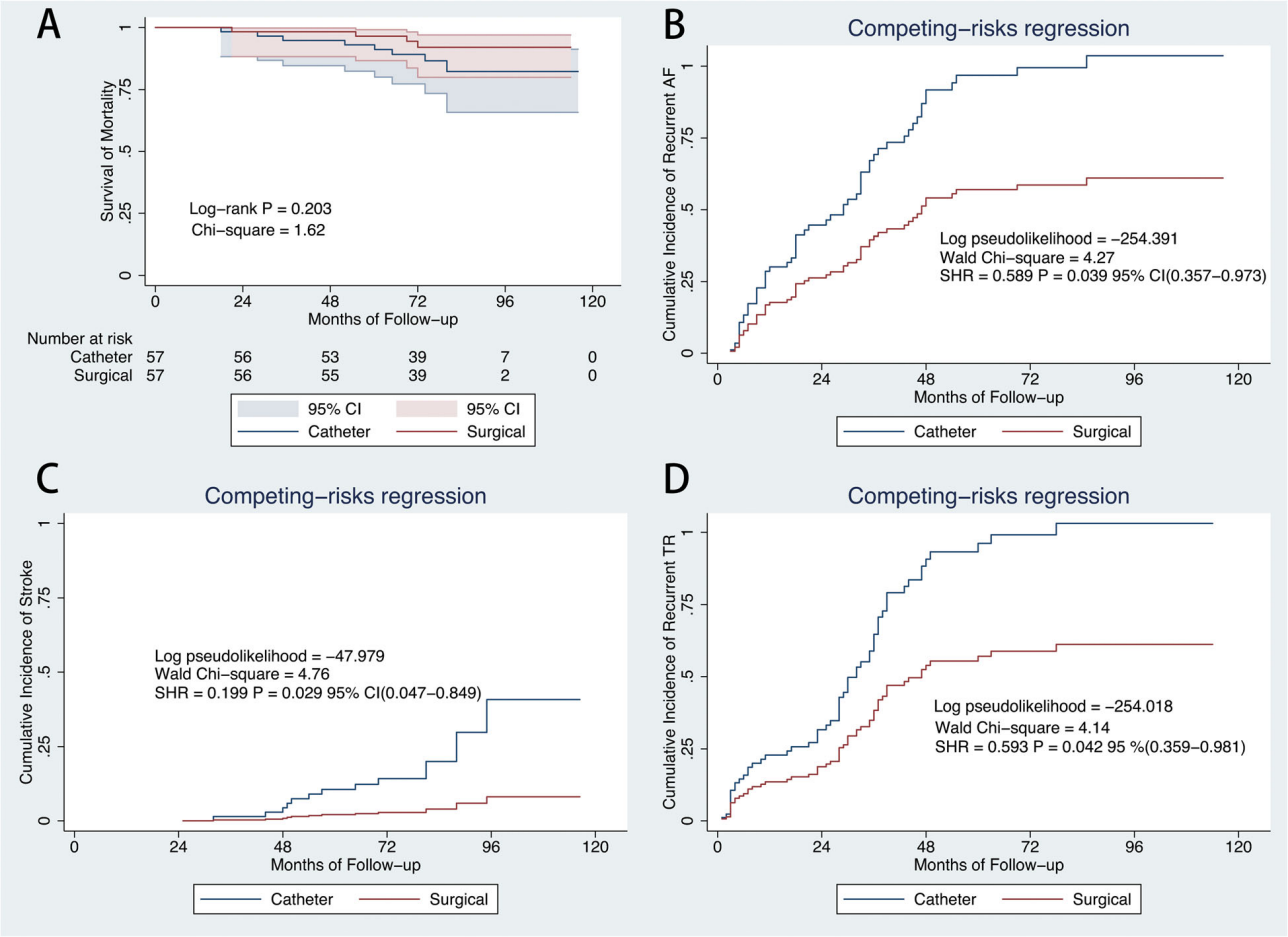
Comparison of outcomes in subgroups with tethering height ≥ 6 mm after PSM. Kaplan–Meier curves showing survival from mortality(**a**). Cumulative incidence curve of atrial fibrillation recurrence (**b**), stroke (**c**), and recurrent moderate-severe tricuspid regurgitation (**d** by competing-risk regression. CI = confidence interval; SHR = sub-hazard ratio

A propensity-score matching was performed to adjust for differences in measured baseline characteristics between 2 cohorts (Table 1, Supplementary Figure 1). Among the 111 PSM pairs, the catheter group showed significantly higher rates of stroke (SHR = 0.286; 95% CI, 0.106–0.768; *P* = 0.013), and AF recurrence (SHR = 0.669; 95% CI, 0.486–0.976; *P* = 0.036) after considering death as a competing risk (Fig. 6). We categorized matched patients into subgroups by tethering height < 6 mm and ≥ 6 mm to determine whether a graded relation exists with tethering height and our further customizing and selecting the best treatment options for the patients with AF-caused moderate-severe TR. On Kaplan–Meier analysis, there were no difference in survival from mortality in patients with tethering height < 6 mm and ≥ 6 mm (Figs. 7a, 8a). On competing risk analysis, the cumulative incidence of stroke, recurrent AF and moderate-severe TR showed no difference between two groups (Fig. 7 b-d). However, the cumulative incidence of stroke (SHR = 0.199; 95% CI, 0.047–0.849; *P* = 0.029), recurrent AF (SHR = 0.589; 95% CI, 0.357–09738; *P* = 0.039) and moderate-severe TR (SHR = 0.593; 95% CI, 0.359–0.981; *P* = 0.042) were higher for patients with tethering height ≥ 6 mm in catheter group (Fig. 8 b-d). Examples of two patients in each cohort with different TR severity at follow-up are shown in Supplementary Figure 2 and 3.

## Discussion

According to our knowledge, this is the first study to compare the mid-term follow-up outcomes of catheter and surgical treatments in patients with LSPAF and moderate-severe functional TR. The main findings showed that (i) catheter ablation without intervention for TV seemed to be an optional treatment in patients with less TV tethering, achieving satisfied SR restored rate and TR improvement; (ii) whereas patients with less leaflets coaptation might benefit more from surgical ablation and TV repair to achieve better SR restored rate and less TR deterioration than catheter ablation.

Gould et al. first suggested that if AF increased the end-diastolic volume above a critical level, then it resulted in TR [17]. Isolated tricuspid annular dilatation that is related to RA enlargement might not be enough to cause severe functional TR in patients with AF, although most of the studies reported that tricuspid annular dilatation is related to RA volume. Further tricuspid annular dilatation with RV enlargement and remodeling might develop into severe TR, leading to an altered ventricular force balance to close the leaflets and incomplete closure of the TVs. It has been reported that RV eccentric dilatation and tethering of leaflets might be more important in patients with severe TR than dilatation of tricuspid annular [18, 19]. These results revealed that TRAF occurred secondary to right-sided heart remodeling, TV deformations and annular dilatation, which was consistent with the present study results. Existing dysfunction or remodeling of RV can act as a substrate for AF occurrence as well. So, AF caused RV remodeling can beget TR, which in turn causes AF further.

The importance of heart rhythm in RV functioning is well known [20, 21]. The RV ejection fraction in patients with AF was deteriorated compared to healthy individuals. AF may lead to RV dysfunction and remodeling, which subsequently caused papillary muscle displacement, tethering of TV leaflet, tricuspid annular dilation, and TR. In our study, we assessed the clinical and echocardiographic parameters that contributed to AF and TR progression after ablation, and the results revealed that RVSI and tethering height were both independent risk factors for the recurrence of either AF or moderate-severe TR. Moreover, ROC analysis suggested that tethering height was a stronger predictor than RVSI in the recurrence of either AF or moderate-severe TR. The higher the basal tethering height was, the more changes in the TR severity at follow-up were, and the less SR restored duration after the procedure was. The results indirectly indicated that right-sided heart remodeling was strongly associated with rhythm status as well as a vicious cycle, thereby leading to worsened TR that progress over time.

Our PSM analysis demonstrates that the cumulative incidence of recurrent AF and stroke in surgical group were lower than those in catheter group. It demonstrated that besides improving efficacy, thromboembolic risk can be further decreased by exclusion of the left atrial appendage. But after categorized matched patients into subgroups by tethering height < 6 mm and ≥ 6 mm, we found that in patients with tethering height < 6 mm, there was no difference in the cumulative incidence of recurrent AF and moderate-severe TR between catheter ablation and surgical treatment. It suggested that that the AF-caused moderate-severe TR could be potentially treated with ‘2C3L’ catheter ablation in patients with tethering height less than 6 mm by a successful restoration of SR. Whereas, as for patients with severe leaflets tethering, the results favored surgical treatment over catheter ablation.

It is remarkable that not all patients with AF-caused moderate-severe TR would benefit from a rhythm control approach for treating AF. Patients with tethering height less than 6 mm who develop TRAF may represent a special category that derives significant clinical benefit from restoration of SR without surgery. This issue would be best answered in a prospective study.

TV surgery is the definitive therapy for most of the patients with severe symptomatic TR [22]. Most of the recommendations are targeted at patients who undergo concomitant left-sided heart procedures [23]. There is a paucity of recommendations regarding surgery in patients with TRAF. Due to lack of outcomes in literature, the current guidelines do not directly address the management of TRAF. TV annuloplasty is widely recommended for the treatment of functional TR [24]. The rationale for the currently used TV annuloplasty techniques is based primarily on the assumption that the major cause of functional TR is annular dilatation. However, recurrent ≥3+ TR after repair occurs in 3–14% of patients with steady increase in its incidence over time and affects up to 20% of patients by 5 years [25, 26]. Severe TR with right-sided heart remodeling frequently presents with tethering of leaflets, which has been addressed as an independent predictive risk factor of recurrent TR [27, 28].

Therefore, simple annuloplasty techniques might not be adequate to correct TR in patients with severe tethering. Dreyfus first introduced a technique by using an autologous pericardial patch to enlarge the anterior tricuspid leaflet to overcome the tethering effects of the dilated RV [29]. It effectively increased the surface of coaptation by three-fold and allowed leaflet coaptation to take place within the RV at the level of tethered septal and posterior leaflets, while maintaining the leaflet mobility. This effectively compensated for severe leaflet tethering, as leaflet coaptation is achieved with reduced leaflet tension [30–32]. Recently, we have used an autologous pericardial patch to enlarge the anterior tricuspid leaflet in patients with tethering height more than 8 mm. (Supplementary Figure 4) But the clinical results requires further investigation in patients with severe TR.

### Study limitations

However, there are several limitations in this study that needs to be acknowledged. Firstly, the relatively limited sample size and inherent retrospective nature of the study inevitably led to missing data and recall bias. Differential losses to follow-up also biased this retrospective cohort study. Secondly, only 2-dimensional echocardiographic parameters were used to evaluate RV geometry and TV tethering. Although 2D echocardiography is widely used in clinical practice and also for research currently, especially for patients with AF, 2D images lacked from inherent limitations such as incomplete comprehension of 3D tricuspid annulus. 3D echocardiography with one beat acquisition might provide more accurate measurements with more reproducible landmarks and could be used to confirm the present findings in further studies [33, 34]. Thirdly, in our subgroup analysis, although propensity-score matching adjusts for baseline imbalances, the analysis does not control for unmeasured confounders. Moreover, small subgroup of patients and the analysis is likely underpowered, a longer-term follow-up results need be validated.

## Conclusions

Our findings suggested that patients with TRAF, has right-sided heart dysfunction and less leaflets coaptation, which may be at risk of recurrence of AF and moderate-severe TR after catheter or surgical treatment. Despite having a moderate or greater degree of TR, patients with TRAF whose tethering height was less than 6 mm showed satisfied improvement in valve function with restoration of SR after catheter ablation. However, in patients with severe leaflets tethering, the results favored surgical treatment over catheter ablation. Since our study is retrospective in nature and whether all patients with TRAF should undergo above-mentioned strategy must be addressed in further prospective study.

## Supplementary information

**Additional file 1: Supplementary Table 1**. Echocardiographic characteristics of catheter cohort at baseline and follow-up. **Supplementary Table 2.** Echocardiographic characteristics of surgical cohort at baseline and follow-up. **S** Figure 1**.** Propensity-score matching for the total cohort. A, Dot plot of patients in either matched or unmatched groups. B, Histograms with overlaid kernel density estimates of standardized differences before and after matching. C, Distribution of propensity scores of surgical (“treated”) and catheter cohort (“control”) before and after matching with overlaid kernel density estimate. D, Line plot of standardized differences before and after matching. E, Dot plot of standardized mean differences (Cohen’s d) for all covariates before and after matching. **S** Figure 2**.** Example of a patient in surgical cohort with a preoperational tethering height of 1.22 cm (A), had a significant moderatesevere TR at 18 months follow-up with recurrent AF (B). **S** Figure 3**.** Example of a patient with a preoperational moderate-severe TR and tethering height of 0.4 cm in catheter cohort (A), had no TR at 24 months follow-up with sinus rhythm (B). **S** Figure 4**.** Example of using an autologous pericardial patch (white arrow) to enlarge the anterior tricuspid leaflet in patients with tethering height more than 8 mm.

## Data Availability

The datasets used and/or analyzed during the current study are available from the corresponding author on reasonable request.

## Abbreviations

AF: Atrial fibrillation
AUC: Area under the ROC curve
CHF: Congestive heart failure
CIs: Confidence intervals
PSM: Propensity-score match
PVAI: Pulmonary vein antrum isolation
RA: Atrial are
ROC: Receiver operating characteristic
RV: Right ventricular
RVFAC: Right ventricular fractional area change
RVSI: Right ventricular sphericity inde
SD: Standard deviation
SR: Sinus rhythm
TAD: Tricuspid annular dimension
TR: Tricuspid regurgitation
TV: Tricuspid valve
VC: Vena contracta
