# Supplementary material for: Catheter ablation or surgical therapy in moderate-severe tricuspid regurgitation caused by long-standing persistent atrial fibrillation. Propensity score analysis
Source: J Cardiothorac Surg. 2020 Sep 29;15:277. doi: 10.1186/s13019-020-01336-3 (PMC7526327; doi:10.1186/s13019-020-01336-3)

SUPPLEMENTAL MATERIAL

Supplementary Table 1. Echocardiographic characteristics of catheter cohort at baseline and follow-up

	Rhythm at Follow-up (SR n =175) (Recurrent AF n = 104)	Baseline	P value (Recurrent AF vs. SR at Baseline)	Follow-up	P value (Baseline vs. Follow-up)	P value (Recurrent AF vs. SR at Follow-up)
LVESD, mm	Sinus	34.6±12.4	0.302	34.8±9.2	0.802	0.620
	Recurrent AF	37.4±11.9		37.9±5.0	0.753	
LVEDD, mm	Sinus	53.9±7.8	0.217	52.6±7.7	0.217	0.129
	Recurrent AF	51.8±6.1		52.7±7.3	0.842	
sPAP, mmHg	Sinus	38.4±6.3	0.292	37.3±8.2	0.370	0.151
	Recurrent AF	39.9±5.2		39.6±6.6	0.524	
LAD, mm	Sinus	53.1±10.6	0.093	53.8±9.7	0.510	0.159
	Recurrent AF	52.8±12.1		54.5±11.2	0.061	
LVEF, %	Sinus	62.1±5.9	0.721	60.4±10.1	0.443	0.999
	Recurrent AF	59.8±7.3		59.4±6.8	0.520	
RAA, mm^2	Sinus	20.1±4.7	0.093	18.3±6.9	0.076	0.040
	Recurrent AF	19.8±5.2		21.3±6.9	0.037	
RVSI	Sinus	2.0±0.6	0.039	2.1±0.2	0.014	<0.001
	Recurrent AF	1.9±0.1		1.8±0.5	0.004	

RVFAC, %	Sinus	44.6±3.9	0.091	45.2±8.9	0.048	0.031
	Recurrent AF	42.9±6.3		40.6±7.2	0.042	
Tethering height, mm	Sinus	4.5±1.5	0.004	3.1±3.6	0.013	<0.001
	Recurrent AF	7.6±1.9		8.6±1.9	0.069	
Tethering area, cm^2	Sinus	1.8±0.7	0.034	1.1±0.4	0.020	<0.001
	Recurrent AF	2.0±0.9		2.6±0.7	0.072	
TAD, cm	Sinus	35.2±3.5	0.077	33.0±6.7	0.016	0.008
	Recurrent AF	36.9±3.7		39.2±6.2	0.006	
TR EROA, cm^2	Sinus	0.7±0.6	0.096	0.4±0.5	0.028	0.015
	Recurrent AF	0.8±0.7		1.0±0.6	0.021	
TR VC, cm	Sinus	0.9±0.8	0.087	0.4±0.3	0.012	0.008
	Recurrent AF	1.0±0.3		1.1±0.5	0.057	
TR Grade	Sinus	3.2±0.3	0.051	2.4±0.8	0.013	<0.001
	Recurrent AF	3.6±0.4		3.9±0.2	0.048	

AF = atrial fibrillation; EROA = effective regurgitant orifice area; LAD = left atrial diameter; LVEF = left ventricle ejection fraction; LVEDD = left ventricle end-diastolic dimension LVESD = left ventricle end-systolic dimension; RAA = right atrial

area; RVFAC = right ventricle fractional area change; RVSI = right ventricle sphericity index; sPAP = pulmonary artery systolic pressure; TAD = tricuspid annulus diameter; TR = tricuspid regurgitation; VC = vena contracta.

Supplementary Table 2. Echocardiographic characteristics of surgical cohort at baseline and follow-up

	Rhythm at Follow-up (SR n =66) (Recurrent AF n = 48)	Baseline	P value (Recurrent AF vs. SR at Baseline)	Follow-up	P value (Baseline vs. Follow-up)	P value (Recurrent AF vs. SR at Follow-up)
LVESD, mm	Sinus	38.1±10.7	0.459	36.8±10.4	0.819	0.115
	Recurrent AF	37.0±13.1		37.9±.6	0.371	
LVEDD, mm	Sinus	54.2±4.9	0.348	53.8±10.1	0.812	0.258
	Recurrent AF	52.9±8.1		53.1±6.9	0.568	
sPAP, mmHg	Sinus	40.5±5.9	0.326	39.8±9.3	0.744	0.721
	Recurrent AF	41.0±7.1		40.6±7.2	0.405	
LAD, mm	Sinus	54.1±9.6	0.096	53.8±10.5	0.184	0.629
	Recurrent AF	53.9±4.3		54.6±9.5	0.333	
LVEF, %	Sinus	57.9±6.4	0.953	59.7±9.6	0.118	0.154
	Recurrent AF	59.2±5.8		60.4±10.0	0.635	
RAA, mm^2	Sinus	21.6±2.7	0.093	20.5±7.4	0.064	0.046
	Recurrent AF	20.1±3.6		22.7±8.1	0.043	
RVSI	Sinus	2.0±0.7	0.020	2.1±0.9	0.012	<0.001
	Recurrent AF	1.9±0.2		1.8±0.7	0.045	
RVFAC, %	Sinus	41.3±8.3	0.087	43.9±7.3	0.028	0.013

	Recurrent AF	42.9±6.8		40.7±9.0	0.024	
Tethering height, mm	Sinus	8.2±3.6	0.007	4.9±3.7	<0.001	<0.001
	Recurrent AF	14.3±4.8		13.9±4.1	0.920	
Tethering area, cm^2	Sinus	3.9±0.3	0.041	1.4±0.9	0.006	<0.001
	Recurrent AF	4.3±0.3		4.6±1.0	0.485	
TAD, cm	Sinus	38.8±6.7	0.059	34.7±5.9	0.001	<0.001
	Recurrent AF	40.1±4.0		43.8±5.7	0.008	
TR EROA, cm^2	Sinus	0.9±0.2	0.098	0.5±0.7	0.003	0.023
	Recurrent AF	1.0±0.6		1.1±0.4	0.089	
TR VC, cm	Sinus	0.9±0.4	0.084	0.5±0.6	0.009	0.002
	Recurrent AF	1.0±0.3		1.1±0.9	0.819	
TR Grade	Sinus	3.4±0.7	0.089	2.7±0.6	0.012	<0.001
	Recurrent AF	3.6±0.6		3.8±0.7	0.812	

AF = atrial fibrillation; EROA = effective regurgitant orifice area; LAD = left atrial diameter; LVEF = left ventricle ejection fraction; LVEDD = left ventricle end-diastolic dimension LVESD = left ventricle end-systolic dimension; RAA = right atrial area; RVFAC = right ventricle fractional area change; RVSI = right ventricle sphericity index; sPAP = pulmonary artery systolic pressure; TAD = tricuspid annulus diameter; TR = tricuspid regurgitation; VC = vena contracta.

S Figure 1. Propensity-score matching for the total cohort. A, Dot plot of patients in either matched or unmatched groups. B, Histograms with overlaid kernel density estimates of standardized differences before and after matching. C, Distribution of propensity scores of surgical (“treated”) and catheter cohort (“control”) before and after matching with overlaid kernel density estimate. D, Line plot of standardized differences before and after matching. E, Dot plot of standardized mean differences (Cohen’s d) for all covariates before and after matching.

ACEI indicates angiotensin-converting enzyme inhibitor; ACT, aortic clamp time; ARB, angiotensin receptor blocker; BMI, body mass index; CAD, coronary artery disease; COPD, chronic obstructive pulmonary disease; EROA, effective regurgitant orifice area; LAD, left atrial diameter; LVEDD, left ventricle end- diastolic dimension; LVEF, left ventricle ejection fraction; LVESD, left ventricle end-systolic dimension; NYHA, New York Heart Association; RA, right atrial; RVFAC, right ventricle fractional area change; RVSI, right ventricle sphericity index; sPAP, pulmonary artery systolic pressure; TAD, tricuspid annulus diameter; TR, tricuspid regurgitation; VC, vena contracta.

S Figure 2. Example of a patient in surgical cohort with a preoperational tethering height of 1.22cm (A), had a significant moderate-severe TR at 18 months follow-up with recurrent AF (B).

S Figure 3. Example of a patient with a preoperational moderate-severe TR and tethering height of 0.4 cm in catheter cohort (A), had no TR at 24 months follow-up with sinus rhythm (B).

S Figure 4. Example of using an autologous pericardial patch (white arrow) to enlarge the anterior tricuspid leaflet in patients with tethering height more than 8mm.

S Figure 1.

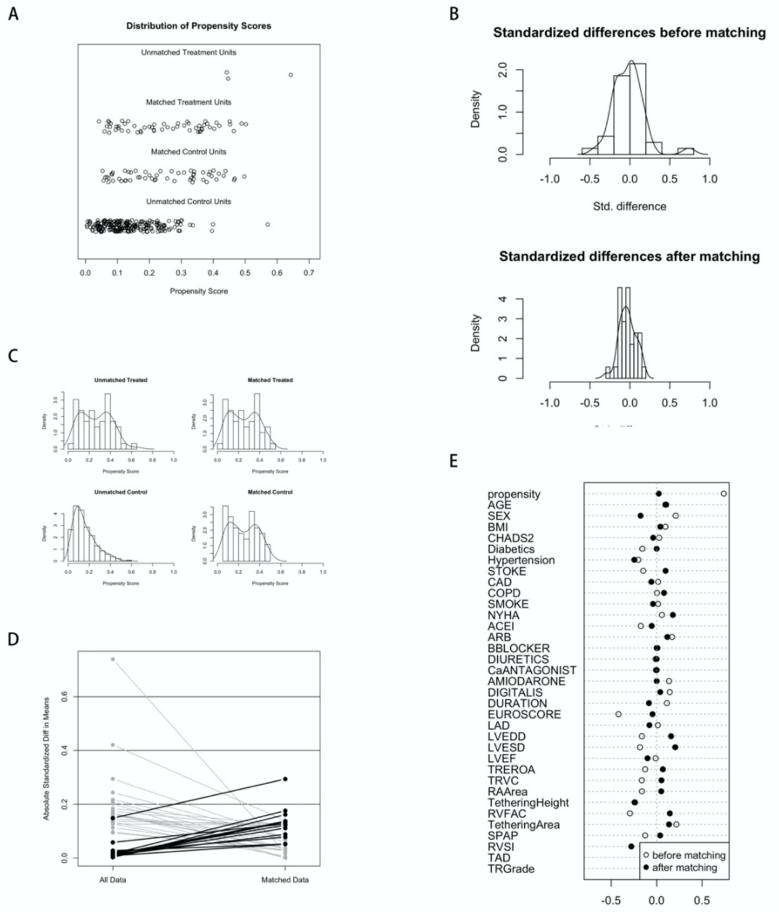
A
Distribution of Propensity Scores
Unmatched Treatment Units
Matched Treatment Units
Matched Control Units
Unmatched Control Units
Propensity Score
B
Standardized differences before matching
Density
Std. difference
Standardized differences after matching
C
Unmatched Treated
Matched Treated
Unmatched Control
Matched Control
D
Absolute Standardized Diff in Means
All Data
Matched Data
E
propensity
AGE
SEX
BMI
CHADS2
Diabetics
Hypertension
STOKE
CAD
COPD
SMOKE
NYHA
ACEI
ARB
BBLOCKER
DIURETICS
CaANTAGONIST
AMIODARONE
DIGITALIS
DURATION
EUROSCORE
LAD
LVEDD
LVESD
LVEF
TREROA
TRVC
RAArea
TetheringHeight
RVFAC
TetheringArea
SPAP
RVSI
TAD
TRGrade
before matching
after matching

S figure 2.

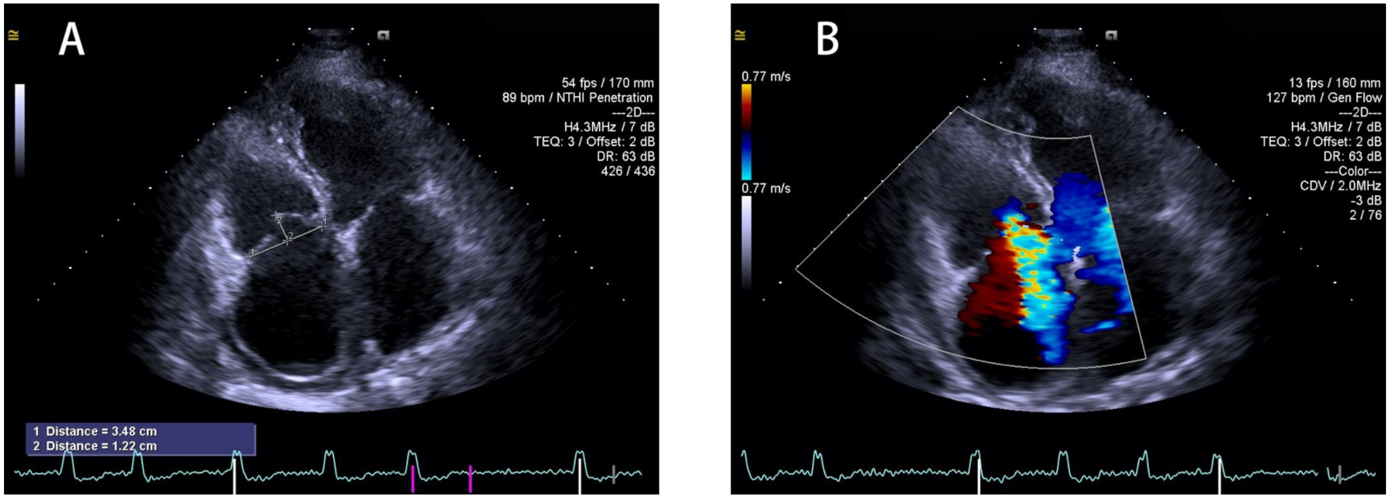
A
54 fps / 170 mm
89 bpm / NTHI Penetration
---2D---
H4.3MHz / 7 dB
TEQ: 3 / Offset: 2 dB
DR: 63 dB
426 / 436
1 Distance = 3.48 cm
2 Distance = 1.22 cm
B
0.77 m/s
0.77 m/s
13 fps / 160 mm
127 bpm / Gen Flow
---2D---
H4.3MHz / 7 dB
TEQ: 3 / Offset: 2 dB
DR: 63 dB
---Color---
CDV / 2.0MHz
-3 dB
2 / 76

S Figure 3.

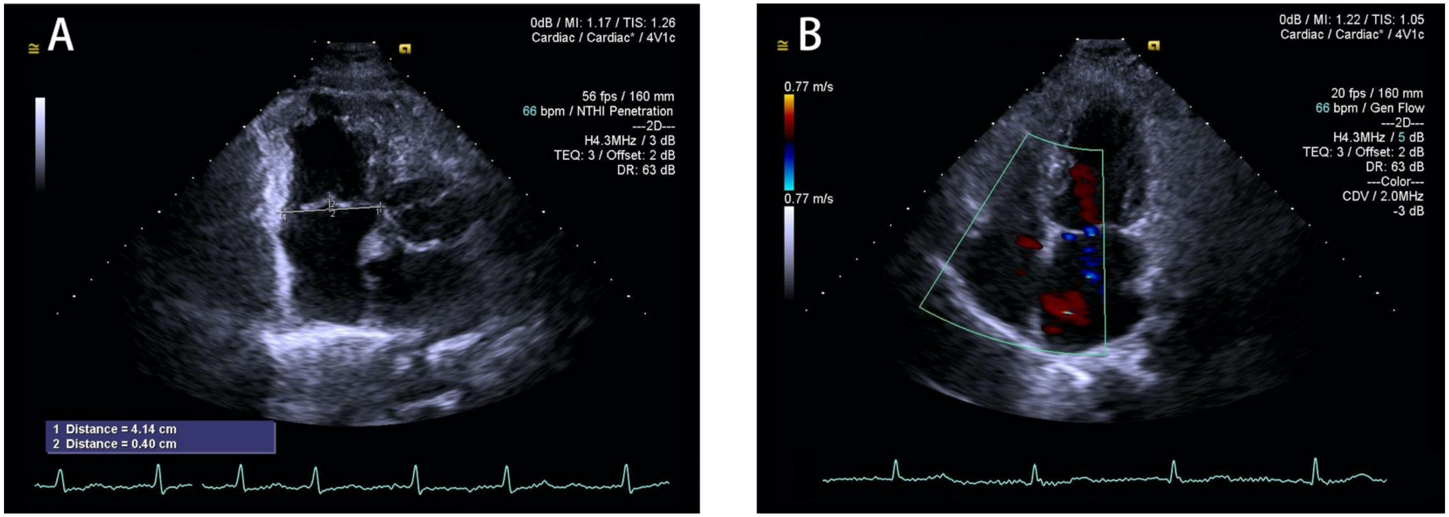
A
0dB / MI: 1.17 / TIS: 1.26
Cardiac / Cardiac° / 4V1c
56 fps / 160 mm
66 bpm / NTHI Penetration
---2D---
H4.3MHz / 3 dB
TEQ: 3 / Offset: 2 dB
DR: 63 dB
1 Distance = 4.14 cm
2 Distance = 0.40 cm
B
0dB / MI: 1.22 / TIS: 1.05
Cardiac / Cardiac° / 4V1c
0.77 m/s
0.77 m/s
20 fps / 160 mm
66 bpm / Gen Flow
---2D---
H4.3MHz / 5 dB
TEQ: 3 / Offset: 2 dB
DR: 63 dB
---Color---
CDV / 2.0MHz
-3 dB

S Figure 4.

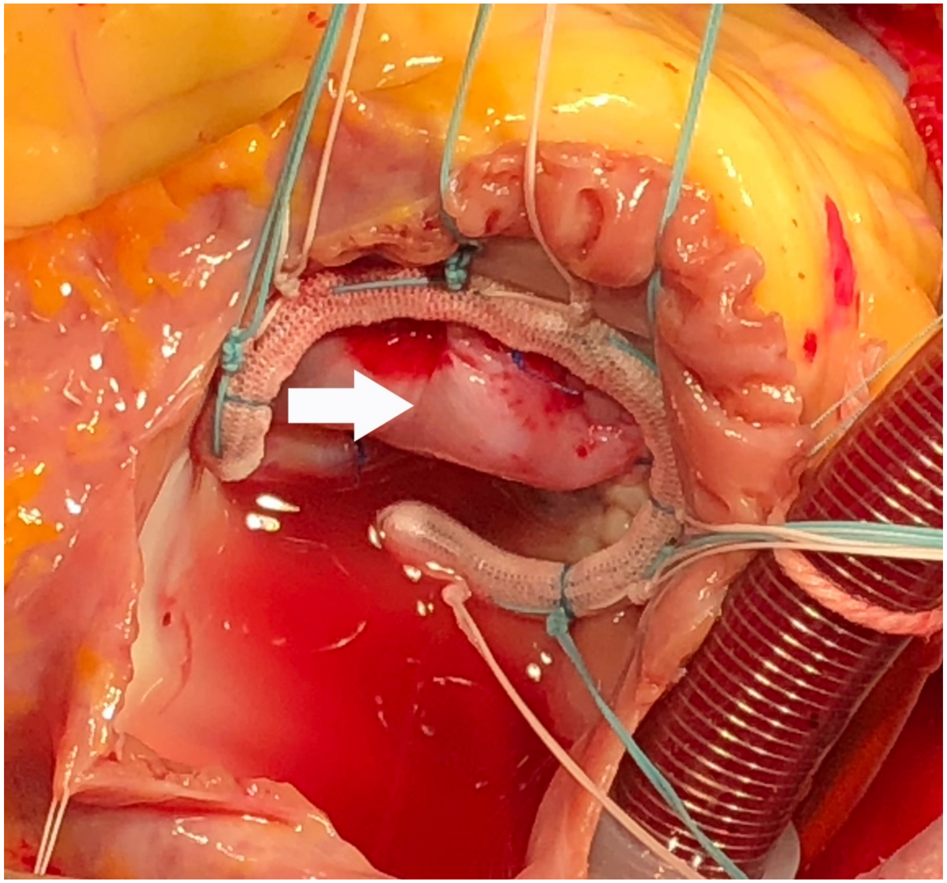

Supplement: Supplementary file 1 — Additional file 1: Supplementary Table 1. Echocardiographic characteristics of catheter cohort at baseline and follow-up. Supplementary Table 2. Echocardiographic characteristics of surgical cohort at baseline and follow-up. S Figure 1. Propensity-score matching for the total cohort. A, Dot plot of patients in either matched or unmatched groups. B, Histograms with overlaid kernel density estimates of standardized differences before and after matching. C, Distribution of propensity scores of surgical (“treated”) and catheter cohort (“control”) before and after matching with overlaid kernel density estimate. D, Line plot of standardized differences before and after matching. E, Dot plot of standardized mean differences (Cohen’s d) for all covariates before and after matching. S Figure 2. Example of a patient in surgical cohort with a preoperational tethering height of 1.22 cm (A), had a significant moderatesevere TR at 18 months follow-up with recurrent AF (B). S Figure 3. Example of a patient with a preoperational moderate-severe TR and tethering height of 0.4 cm in catheter cohort (A), had no TR at 24 months follow-up with sinus rhythm (B). S Figure 4. Example of using an autologous pericardial patch (white arrow) to enlarge the anterior tricuspid leaflet in patients with tethering height more than 8 mm. [file 13019_2020_1336_MOESM1_ESM.pdf]
